# Diffuse Alveolar Hemorrhage Caused by Warfarin after Rifampicin Discontinuation

**DOI:** 10.1155/2019/4917856

**Published:** 2019-01-23

**Authors:** Takashige Kiyota, Seiji Shiota, Ryosuke Hamanaka, Daisuke Tsutsumi, Takeshi Takakura, Eishi Miyazaki

**Affiliations:** ^1^Department of General Medicine, Almeida Memorial Hospital, Oita 870-1195, Japan; ^2^Department of General Medicine, Oita University Faculty of Medicine, Yufu 879-5593, Japan

## Abstract

An 83-year-old man under warfarin therapy presented for assessment of prolonged prothrombin time and cough. High-resolution computed tomography findings of the chest showed diffuse alveolar hemorrhage. His international normalized ratio (INR) was 11.89. He had been treated with rifampicin for a persistent infection, but this had been discontinued about two months before admission. Rifampicin suppresses the anticoagulant activity of warfarin, which can lead to a need for increased doses of warfarin to achieve and maintain a therapeutic INR. More frequent INR monitoring is needed even after discontinuing rifampicin.

## 1. Introduction

Warfarin has been widely applied for anticoagulation during thromboembolic complications [1]. Monitoring the international normalized ratio (INR) is important to reduce the risk of bleeding during warfarin therapy. However, many drug interactions influence the anticoagulant effects of warfarin. Many medications, including warfarin, are metabolized by the cytochrome P450 (CYP) oxidative enzyme system, whereas rifampicin and others induce this system. These different pathways can result in serious drug interactions. Here, we describe an 83-year-old patient under warfarin therapy who developed diffuse alveolar hemorrhage after discontinuing rifampicin.

## 2. Case Presentation

An 83-year-old man was referred to for assessment of prolonged prothrombin time and cough. His medical history included atrial fibrillation, hypertrophic cardiomyopathy, vasospastic angina, osteoarthritis of the hip, and total hip arthroplasty followed by infection. He presented with mild respiratory distress. His vital signs were as follows: temperature of 36.5°C, irregular pulse of 107 bpm, respiratory rate of 12 per minute, blood pressure of 119/63 mmHg, and oxygen saturation of 89% on room air. He had normal first and second heart sounds, diffuse rhonchi over both lung fields, and purpura on the lateral surface of the left thigh and the medial surface of the right knee. Evidence of other bleeds including petechial, ecchymosis, epistaxis, and gastrointestinal bleeding was not found.

The patient's medications on the day of admission were warfarin (6 mg/day), bisoprolol (2.5 mg/day), ubidecarenone (30 mg/day), benidipine (8 mg/day), nicorandil (10 mg/day), and imidapril (5 mg/day). He had a long-term MRSA infection that had been treated with rifampicin for four years, but this had been discontinued about two months before admission to our hospital. His most recent INR was 3.2 at six weeks before admission. He had no dementia and good compliance with medication.

Laboratory findings on admission were as follows: WBC 5,280/*μ*L, hemoglobin 9.0 g/dL, hematocrit 27.4%, platelets 145,000/*μ*L, INR 11.89, PT 146.6 s, APTT 99.6 s, D-dimer 1.1 *μ*g/mL, random glucose 102 mg/dL, serum sodium 139 mEq/L, serum potassium 4.0 mEq/L, and serum creatinine 0.96 mg/dL. Urinalysis showed macroscopic hematuria. All other laboratory findings including liver function were unremarkable. Serum C-ANCA and P-ANCA were negative. Arterial blood gas analysis indicated a pH of 7.426, partial pressure of carbon dioxide in arterial blood (PaCO_2_) 37.9 mmHg, partial pressure of arterial oxygen (PaO_2_) 76.7 mmHg, and bicarbonate (HCO_3_^−^) 24.5 mmol/L with 28% oxygen inhalation via a nasal cannula.

**Figure 1:**
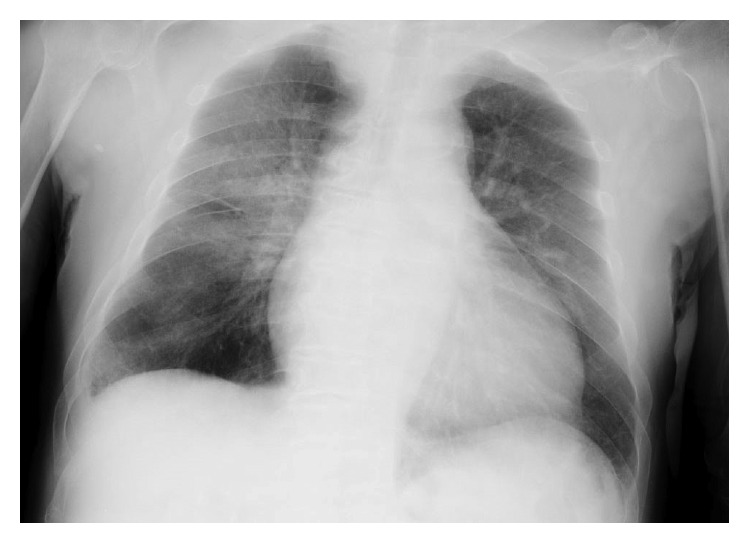
Chest X-ray findings on admission. Image shows alveolar opacity in both lungs, particularly in upper and middle lobes.

**Figure 2:**
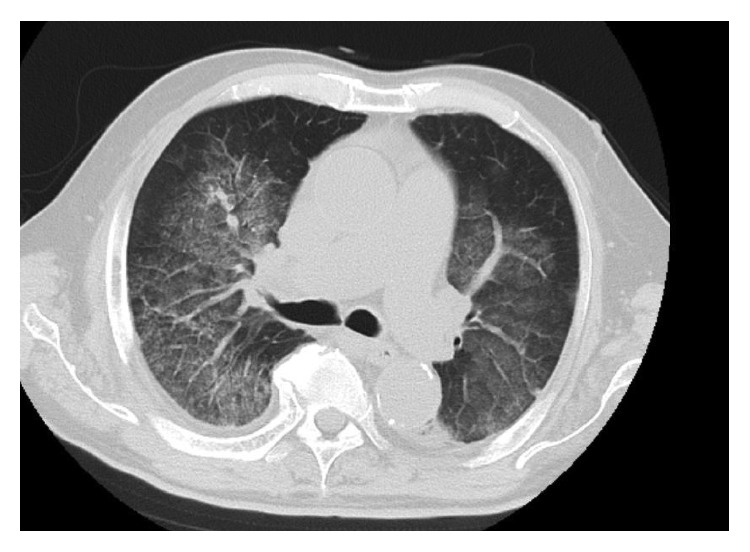
Chest computed tomography findings on admission. High-resolution computed tomography of the chest shows reticular opacity with bronchial-wall thickening and interlobular septal thickening in both lung fields.

Chest radiography revealed alveolar opacity in both lungs, especially in the upper and middle lobes (Figure 1). High-resolution computed tomography of the chest confirmed reticular opacity with bronchial wall thickening and interlobular septal thickening in both lungs (Figure 2). Bronchoalveolar lavage samples indicated alveolar hemorrhage. He had no history or recurrence of urothelial infection and no evidence of urothelial malignancy.

The high INR (11.89) indicated that the main cause of the diffuse alveolar hemorrhage and macroscopic hematuria was excessive warfarin anticoagulant activity. Therefore, warfarin was discontinued on the day of admission, and vitamin K 10 mg, carbazochrome sodium sulfonate 50 mg, tranexamic acid 1 g, and four units of fresh frozen plasma (FFP) were administered. The INR decreased to 1.46 on the following day, and oxygen saturation in room air gradually improved from 89% to 93% after admission. Chest radiography on days 6 and 12 revealed decreased ground glass opacity, and high-resolution computed tomography showed improvements in the lung fields. The patient was discharged from hospital on day 13.

## 3. Discussion

Diffuse alveolar hemorrhage is a life-threatening clinical syndrome caused by various diseases. Diffuse alveolar hemorrhage due to warfarin therapy has been described [2–4]. Warfarin is a racemic mixture of S and R enantiomers that are metabolized in different ways in the liver. However, most of them are metabolized by CYP oxidative enzymes. About 90% of the S enantiomer is oxidatively metabolized mainly by CYP2C9 and, to a lesser extent, by CYP3A4. About 60% of the R enantiomer is oxidatively metabolized mainly by CYP1A2 and CYP3A4 and, to a lesser extent, by CYP2C19 [5]. The S enantiomers of warfarin have about 3∼5-fold more anticoagulant activity than the R enantiomers.

This patient had undergone a total left hip arthroplasty about 10 years ago and developed an infection about four years postoperatively that had been treated with rifampicin for the past four years. However, rifampicin was discontinued about two months before admission to our hospital. Rifampicin is used to treat device-related infections, and it is a potent inducer of the hepatic CYP oxidative enzyme system [6]. The coadministration of rifampicin and other drugs that are metabolized by the CYP oxidative enzyme system can result in serious interactions. When rifampicin and warfarin are concurrently administered, rifampicin induces the CYP2C9, CYP3A4, CYP1A2, and CYP2C19 enzymes that accelerate clearance of the warfarin R and S enantiomers [7, 8]. Therefore, rifampicin suppresses the anticoagulant activity of warfarin, which can cause a need to increase the warfarin dose to achieve and maintain a therapeutic INR [9]. In fact, several reports describe that warfarin dose is increased among patients administered with concurrent rifampin [6, 10, 11]. A two-to three-fold increase in the warfarin dose might be necessary during concurrent therapy to account for this drug-drug interaction [12]. The present patient had been maintained on warfarin at 2.0 mg/day before starting rifampicin, and then the warfarin dose was tripled. Pediatric patients under treatment with rifampicin are at risk for an attenuated response to warfarin [13]. When rifampicin is discontinued, clearance of the S and R enantiomers of warfarin normalizes, which leads to high blood concentrations and excessive warfarin anticoagulant activity. One week to several months after discontinuation is necessary for the enzyme-inducing effects of rifampin to fully subside [6].

Martins et al. reported that a hyperanticoagulation state of warfarin can occur after rifampicin discontinuation, resulting in macroscopic hematuria and a need for an immediate reduction in the warfarin dosage [9]. They recommend monitoring patients weekly after stopping rifampicin, until the maintenance dose of warfarin has decreased to that administered before starting rifampicin. Non-vitamin K oral anticoagulants (NOACs) are becoming more frequently applied than warfarin to reduce major bleeding. Our patient started on an NOAC after diffuse alveolar hemorrhage. However, a recent report has described that compared with an NOAC alone, concurrent rifampicin is associated with increased risk of major bleeding among patients under treatment with an NOAC for nonvalvular atrial fibrillation [14]. Therefore, the potential risks of bleeding must be considered if our patient is started on rifampicin again.

In conclusion, the present report describes a rare example of diffuse alveolar hemorrhage caused by warfarin after rifampicin discontinuation. Physicians should be aware of potential drug interactions when starting a patient on a drug in addition to warfarin. On the other hand, anticoagulation activity can be changed even after stopping some agents, particularly rifampicin. More frequent INR monitoring is needed to avoid serious bleeding complications even after rifampicin discontinuation.
